# Increased expression of ALCAM/CD166 in pancreatic cancer is an independent prognostic marker for poor survival and early tumour relapse

**DOI:** 10.1038/sj.bjc.6605136

**Published:** 2009-07-14

**Authors:** C Kahlert, H Weber, C Mogler, F Bergmann, P Schirmacher, H G Kenngott, U Matterne, N Mollberg, N N Rahbari, U Hinz, M Koch, M Aigner, J Weitz

**Affiliations:** 1 Department of Surgery, University of Heidelberg, Im Neuenheimer Feld 110, 69120 Heidelberg, Germany; 2 Institute of Pathology, University of Heidelberg, Im Neuenheimer Feld 110, 69120 Heidelberg, Germany; 3 Institute of Occupational and Social medicine, Voßstrasse 2, 69115 Heidelberg, Germany

**Keywords:** ALCAM, CD166, ADAM17/TACE, pancreatic cancer, prognostic marker

## Abstract

**Background:**

ALCAM (activated leucocyte cell adhesion molecule, synonym CD166) is a cell adhesion molecule, which belongs to the Ig superfamily. Disruption of the ALCAM-mediated adhesiveness by proteolytic sheddases such as ADAM17 has been suggested to have a relevant impact on tumour invasion. Although the expression of ALCAM is a valuable prognostic and predictive marker in several types of epithelial tumours, its role as a prognostic marker in pancreatic cancer has not yet been reported.

**Methods:**

In this study, paraffin-embedded samples of 97 patients with pancreatic cancer undergoing potentially curative resection were immunostained against ALCAM, ADAM17 and CK19. Expression of ALCAM and ADAM17 was semiquantitatively evaluated and correlated to clinical and histopathological parameters.

**Results:**

We could show that in normal pancreatic tissue, ALCAM is predominantly expressed at the cellular membrane, whereas in pancreatic tumour cells, it is mainly localised in the cytoplasm. In addition, univariate and multivariate analyses show that increased expression of ALCAM is an adverse prognostic factor for recurrence-free and overall survival. Overexpression of ADAM17 in pancreatic cancer, however, failed to be a significant prognostic marker and was not coexpressed with ALCAM.

**Conclusions:**

Our findings support the hypothesis that the disruption of ALCAM-mediated adhesiveness is a relevant step in pancreatic cancer progression. Moreover, ALCAM overexpression is a relevant independent prognostic marker for poor survival and early tumour relapse in pancreatic cancer.

Pancreatic cancer is one of the most aggressive tumours in humans. It is the fourth leading cause of cancer death in females and the fifth leading cause in males worldwide (Jemal *et al*, 2008). Despite some progress in the medical treatment, surgical resection remains the only chance for cure in localised stages. However, even in the small subset of approximately 15–20% of patients with pancreatic cancer, who are eligible to curative resection, cancer-related death is still very high. Currently, lymph node status is one of the most important independent prognostic markers in patients who undergo resection (Geer and Brennan, 1993; Delcore *et al*, 1996; Hellan *et al*, 2008). Further clinical and pathological prognostic parameters are tumour typing, differentiation grade and size (Geer and Brennan, 1993); however, this information is currently not used for clinical management of the patients. Adjuvant chemotherapy is commonly offered to all patients with resected pancreatic cancer (Neoptolemos *et al*, 2001); however, only a certain subgroup of patients will benefit from this treatment. Better definition of the patient's individual prognosis might allow a more individualised adjuvant therapy. Therefore, in recent years, the focus has been shifted to define new molecular prognostic markers to gain such prognostic information and to select patient subgroups that might benefit from specific treatment algorithms.

ALCAM (activated leucocyte cell adhesion molecule, synonym CD166) belongs to the Ig superfamily and mediates both heterophilic (ALCAM-CD6) and homophilic (ALCAM-ALCAM) cell–cell interactions (Swart, 2002). It is considered a prognostic marker in melanoma (van Kempen *et al*, 2000), prostate cancer (Kristiansen *et al*, 2005), breast cancer (King *et al*, 2004; Weichert *et al*, 2004; Burkhardt *et al*, 2006), colorectal cancer (Weichert *et al*, 2004), bladder cancer (Tomita *et al*, 2003), oesophageal squamous cell carcinoma (Verma *et al*, 2005) and ovarian cancer (Mezzanzanica *et al*, 2008). However, these data are quite inconsistent. In some tumour types membranous expression is associated with worse prognosis (Weichert *et al*, 2004), whereas in other tumour types cytoplasmic localisation of ALCAM is an adverse prognostic parameter (Burkhardt *et al*, 2006). In addition, increased (Tomita *et al*, 2003; Weichert *et al*, 2004; Kristiansen *et al*, 2005; Verma *et al*, 2005; Burkhardt *et al*, 2006) as well as reduced (King *et al*, 2004; Mezzanzanica *et al*, 2008) expression of ALCAM has been correlated with poor prognosis, depending on the tumour type.

Recently, Bech-Serra *et al* (2006) have shown that ADAM17 acts as a proteolytic sheddase of ALCAM. Rosso *et al* (2007) observed that this process is involved in the motility of epithelial ovarian carcinoma cells. They postulated that the disruption of the ALCAM-mediated adhesiveness is a relevant step, which increased the invasiveness of tumour cells.

The aim of our study was to evaluate the prognostic significance of ALCAM in pancreatic cancer. In addition, based on the observations of Bech-Serra *et al* and Rosso *et al*, we analysed its correlation with ADAM17.

Here, we show that overexpression of ALCAM is an adverse prognostic factor for recurrence-free and overall survival (OS) in pancreatic cancer. However, despite its important role in tumour invasion and progression in pancreatic cancer (Ringel *et al*, 2006) and other types of tumours (Arribas *et al*, 2006; Mochizuki and Okada, 2007), immunohistochemical expression of ADAM17 correlates significantly neither with clinical and pathological data nor with ALCAM expression in pancreatic cancer.

## Materials and methods

### Patients

Paraffin-embedded tumour samples were analysed from 97 patients aged 31–82 years (mean 63, median 65; 44 female, 53 male), who were diagnosed with a primary ductal adenocarcinoma of the pancreas and who underwent tumour resection at the Department of Surgery, University Hospital Heidelberg between 2002 and 2005. The median follow-up period was 18.3 months. A written informed consent had been obtained before resection from all patients regarding tissue sampling; the tissue sampling and the analyses regarding potential prognostic markers were approved by the local ethics committee. No neoadjuvant radio- or chemotherapy was applied before surgical resection to any patient. A total of 60 patients underwent a pylorus-sparing pancreatico-duodenectomy, 11 patients a classic pancreatico-duodenectomy, 17 patients a distal pancreatectomy and 9 patients a total pancreatectomy. According to the AJCC classification, there were 18 patients with tumour stage II, 74 patients with tumour stage III and 5 patients with tumour stage IV. In total, 88 cases were resected R0 and 9 cases were resected R1. Patients with R2 resections were not included in this study. It is to be noted that as all patients included in this study were operated between 2002 and 2005, evaluation of the R status had been still carried out by nonstandardised protocols and not according to the recently suggested new standardised protocols, revealing a significantly higher number of R1 resections (Esposito *et al*, 2008).

After resection 70 patients received adjuvant chemotherapy. This included 35 patients treated with gemcitabine, 22 treated with 5-fluorouracil and 13 subjected to a combined radiochemotherapy. In total, 19 patients did not receive any adjuvant treatment; in 8 cases the type of postoperative treatment was unknown.

### Immunohistochemistry

Comparative studies of ALCAM, ADAM17 and CK19 were carried out on sequential serial sections. Immunohistochemical staining was carried out on 4-*μ*m sections of formalin-fixed, paraffin-embedded tumour samples. The archival tissue array blocks were freshly cut, sections were mounted on SUPERFROST PLUS microscope slides (Menzel, Braunschweig, Germany) and incubated at 37°C overnight. Sections were deparaffinised in xylene, rehydrated in a graded series of ethanol and washed with 1 mol l^−1^ phosphate-buffered saline (PBS). For heat-induced antigen retrieval for ALCAM and ADAM17, slides were boiled in a microwave oven for 5 min (pH 6.0, 0.94 ml Antigen Unmasking Solution per 100 ml distilled water (Vector Laboratories, Inc., Burlingame, CA, USA)) thrice and allowed to cool down at room temperature for at least 20 min. Antigen-retrieval for CK19 staining was achieved by preincubating samples with Pronase E for 5 min. After immersing slides in a 3.0% hydrogen peroxidase solution in methanol for 20 min to inhibit endogenous peroxidase activity, nonspecific binding sites were blocked by preincubation with 10% normal goat serum (Vector Laboratories) in 1 mol l^−1^ PBS for 30 min at room temperature for ALCAM- and CK19 staining. Nonspecific binding in sections for ADAM17 staining was blocked using an Avidin/Biotin Blocking Kit (Vector Laboratories). Slides were incubated overnight at 4°C with primary mouse monoclonal anti-ALCAM antibody (clone MOG/07; Novocastra, Newcastle upon Tyne, UK) at a dilution of 1 : 100 or with rabbit polyclonal anti-ADAM17 antibody (Anti-TACE (807–823); Calbiochem, La Jolla, CA, USA) at a dilution of 1 : 50 in a dilution buffer (Antibody Diluent; Dako A/S, Glostrup, Denmark). Mouse monoclonal anti-human Cytokeratin 19 (clone RCK108; DakoCytomation A/S) was diluted 1 : 100 in dilution buffer and incubated at room temperature for 1 h. To eliminate nonspecific staining resulting from endogenous avidin–biotin activity, slides were incubated with EnVision+ System (DakoCytomation A/S) (EnVision anti-mouse for ALCAM- and CK19-, anti-rabbit for ADAM17 staining) for 30 min at room temperature. Immunoreactions were developed and target antigens were detected using AEC Substrate Chromogen (DakoCytomation A/S) according to the instructions of the manufacturer. Finally, sections were counterstained with haematoxylin, dehydrated in graded concentrations of ethanol and mounted. The staining intensity of each slide was scored as absent: 0, weak: 1, medium: 2 and strong: 3 as described before (Kristiansen *et al*, 2003) on a blind basis by three independent researchers (CK, HW, NM) and two pathologists (CM, FB). Besides, for ALCAM, clinical specimen were characterised as membranous-positive or as non-membranous staining.

### Statistical analysis

*χ*^2^-test were used to examine the statistical significance of ALCAM- and ADAM17 expression in pancreatic cancer in comparison with clinical and pathological parameters. Spearman's correlation test was employed to analyse a correlated expression between ALCAM and ADAM17. Univariate survival analysis was assessed using the Kaplan–Meier method. Differences in survival curves were calculated with the log-rank test. Multivariable analysis (Cox proportional hazards regression model) of OS was carried out on all covariates that showed significant association with OS in univariate analysis. *P*-values of all statistical tests were two-sided and *ρ*<0.05 was considered significant. All statistics were compiled using the software package SPSS, version 11.0 (SPSS, Chicago, IL, USA).

## Results

### Evaluation of specifity of ALCAM and ADAM17 immunohistochemistry

To validate the specificity of immunohistochemical staining against ALCAM, we repeated immunohistochemistry in 15 representing clinical specimen with two different antibodies against ALCAM (primary rabbit polyclonal anti-ALCAM antibody, Atlas Antibodies AB, Stockholm, Sweden and primary mouse monoclonal anti-ALCAM antibody (clone MOG/07; Novocastra)). When comparing the immunohistochemical results of the monoclonal antibody and the polyclonal antibody in sequential slides, the expression pattern were identical in 15/15 samples, though the staining intensity of the polyclonal antibody was weaker than the staining intensity of the monoclonal antibody (Supplementary Figure 4a–g). These results confirm the specifity of the anti-ALCAM staining. For immunohistochemical evaluation of all 97 clinical specimen, we used only the monoclonal antibody (clone MOG/07), which has been successfully approved before (Weichert *et al*, 2004; Mezzanzanica *et al*, 2008).

Likewise, we carried out immunohistochemistry with two different antibodies against ADAM17 (clone 1F6, Abnova, Taiwan and Anti-TACE (807–823); Calbiochem) in 15 representing clinical specimen. In these 15 representing clinical specimen, the expression pattern of ADAM17 was similar by the monoclonal antibody and the polyclonal antibody when compared in sequential slides (Supplementary Figure 5a–f). Particularly, in none of the samples stained against ADAM17, we could detect a membranous expression of ADAM17, as it has been described before (Ringel *et al*, 2006).

For immunohistochemical evaluation of all 97 clinical specimens against ADAM17, we used only the polyclonal antibody, as it displayed a more intense staining warranting a more diligent analysis.

### ALCAM displays a differential cellular expression pattern in pancreatic cancer and normal pancreatic tissue

**Figure 1:**
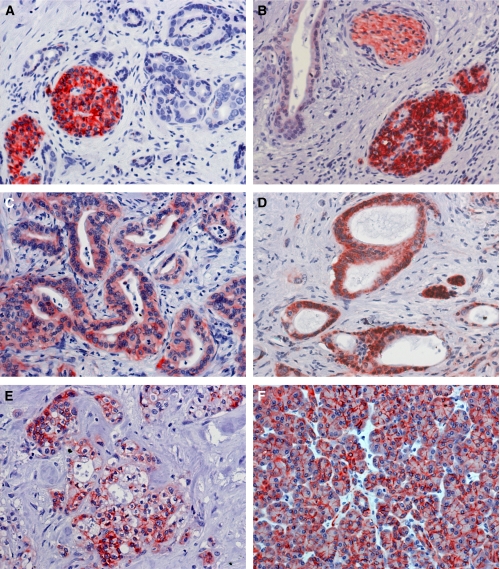
CD166 expression in pancreatic cancer and normal pancreatic tissue. (**A**) Pancreatic tumour cells displaying a CD166 intensity of 0 next to remnant pancreatic islet cells with an intensity of 3. (**B**) Pancreatic tumour cells displaying a CD166 intensity of 1 next to a nerve with a CD166 intensity of 2 and remnant pancreatic islet cells with an intensity of 3. (**C**) Pancreatic tumour cells displaying a CD166 intensity of 2. (**D**) Pancreatic tumour cells displaying a CD166 intensity of 3 next to remnant pancreatic islet cells with an intensity of 3. (**E**) Pancreatic tumour cells displaying membranous expression of CD166. (**F**) Normal pancreatic tissue with membranous staining. Original magnification, × 400.

In pancreatic cancer, 56 samples were ALCAM-positive, 41 samples were ALCAM-negative. It is noted that even in specimen, where immunostaining was absent in tumour cells, endocrine pancreatic islet cells and nerves were always positive. These structures served as an internal control. Endocrine islet cells usually revealed a staining intensity of 3, nerves basically showed a staining intensity of 2 (Figure 1B). Distribution of staining intensity in pancreatic cancer was as follows: Intensity 0: 41 cases, intensity 1: 26 cases, intensity 2: 23 cases and intensity 3: 7 cases. In total, 50 cases (51%) revealed a cytoplasmic expression exclusively (Figure 1A–D, Supplementary Figure 1c). In addition to cytoplasmic expression, membranous positive expression could be focally detected in six specimens (7%) (Figure 1E). In total, four of these samples revealed a staining intensity of 2 and two were scored with a staining intensity of 1. Peritumoral, non-neoplastic pancreatic tissue was present on the examined slides in 60 samples adjacent to pancreatic cancer. In 48 of these samples ALCAM was homogenously expressed in the acinar epithelium (Figure 1F); it is noted that in all of these samples ALCAM was expressed in the cellular membrane. None of the samples of normal pancreatic tissue showed cytoplasmic staining of ALCAM. The differential cellular distribution of ALCAM between pancreatic cancer and corresponding normal pancreatic tissue was highly significant (*P*=0.0001, Table 1).

### ADAM17 immunostaining in normal pancreatic tissue and in pancreatic cancer

**Figure 2:**
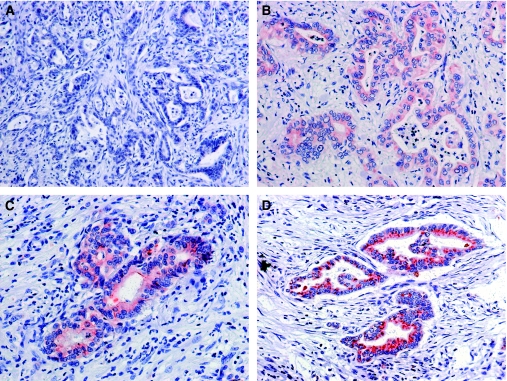
ADAM17 expression in pancreatic cancer. (**A**) Pancreatic tumour cells displaying an ADAM17 intensity of 0. (**B**) Pancreatic tumour cells displaying an ADAM17 intensity of 1. (**C**) Pancreatic tumour cells displaying an ADAM17 intensity of 2. (**D**) Pancreatic tumour cells displaying an ADAM17 intensity of 3. Original magnification, × 400.

A total of 71 pancreatic cancer samples were positive for ADAM17 and 26 samples were negative. In tumour cells, ADAM17 was solely localised in cytoplasm. Adjacent to pancreatic cancer, we occasionally observed positive endocrine pancreatic islet cells, displaying usually an intensity strength of 3. For pancreatic cancer, staining intensities of ADAM17 were scored as intensity 0: 27 cases, intensity 1: 16 cases, intensity 2: 40 cases and intensity 3: 14 cases (Figure 2A–D, Supplementary Figure 2b–d). In normal pancreatic tissue, 59 out of 60 cases were scored as negative. This aberrant expression of ADAM17 in pancreatic cancer compared with normal pancreatic tissue was highly significant (*P*<0.0001).

### Co-expression analysis between ALCAM and ADAM17

Previous data have shown that ADAM17 is an important sheddase of ALCAM. Thus, we intended to assess a co-expression between ADAM17/TACE and ALCAM in pancreatic cancer and to evaluate a potential co-function. However, diligent immunohistochemical comparison of sequential sections revealed no significant positive or negative correlated co-expression. When comparing the expression intensities (low/medium/high) or the expression patterns (membranous/cytoplasmic) with each other, statistical analyses neither showed significant correlation (Spearman's correlation coefficient 0.155 *P*=0.128 nor contingence coefficient 0.057 *P*=0.58).

### ALCAM in correlation to clinical and pathological parameters and prognosis

**Figure 3:**
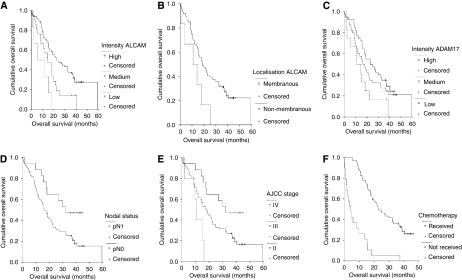
Univariate analysis (log-rank test, Kaplan–Meier curves) of prognostic parameters in pancreatic cancer. (**A**) Intensity of cytoplasmic expression of CD166 (*P*=0.0006), (**B**) Membranous *vs* non-membranous expression of CD166 (*P*=0.03), (**C**) Intensity of cytoplasmic expression of ADAM17 (*P*=0.09), (**D**) Nodal status (*P*=0.02), (**E**) AJCC tumour stage (*P*=0.007), (**F**) adjuvant chemotherapy received *vs* not received (*P*=0.0001).

For statistical analysis, samples with ALCAM immunostaining were grouped in low (0, 1), medium (2) and high (3) intensity levels of expression. When comparing the intensity levels to clinical and pathological parameters, there was a significant inverse correlation between the grade of intensity and the survival status of patients after 36 months (*χ*^2^ *P*=0.03, Table 2). There was no significant correlation with age, gender, nodal status, grading, AJCC tumour stage or resection status (Table 2). When analysing prognostic factors for progression-free and OS, univariate analysis using the log-rank test confirmed well-known prognostic parameters such as lymph node status (*P*=0.02), AJCC tumour stage (*P*=0.007) or treatment by adjuvant chemotherapy (*P*=0.0001) (Figure 3D–F Table 3) to be of prognostic relevance in our patient cohort. Regarding ALCAM expression, median OS was significantly reduced in tumours with high intensity level compared with tumours with medium or low intensity expression levels, (high expression: median OS 5.6 months, medium expression: median OS 14.8 months and low expression: median OS 22.8 months, *P*=0.0006, Figure 3A, Table 3). In the same line, progression-free survival was significantly shorter in tumours displaying high levels of ALCAM in comparison with medium- and low-expressing tumours (median progression-free survival times of 7, 8 and 17 months for high, medium and low expression, respectively, *P*=0.008, Table 3). In a further sub-group analysis, the six patients with partial membranous expression had a significant shorter median overall (median: 10.9 *vs* 18.7 months, *P*=0.03) and tumour recurrence-free survival (median: 6 *vs* 15 months, *P*=0.02) compared with tumours with non-membranous ALCAM staining (Figure 3B, Table 3). In a multivariate analysis based on the Cox proportional hazards regression model, we tested the independent predictive value for all relevant clinical and pathological parameters, adjuvant chemotherapy and ALCAM expression. As we used a non-categorial regression model, we compared low-ALCAM intensity expression to medium/high-ALCAM intensity expression. In this analysis, increased ALCAM intensity expression proved to be an independent prognostic marker for OS (*P*=0.0001) in addition to adjuvant treatment of chemotherapy (*P*=0.0001) and tumour stage (*P*=0.007) (Table 4). Lymph node status was excluded for its linear depending covariance with tumour stage.

### ADAM17 in correlation to clinical and pathological parameters and prognosis

In analogy to ALCAM expression, we evaluated immunhistochemical staining of ADAM17 expression in pancreatic cancer. Intensity of staining was scored as low (0, 1), medium (2) and high (3) expression level and compared with clinical and pathological parameters. By crosstable-calculation (*χ*^2^-test), we found no significant correlation with age, nodal status, grading, resection status or AJCC tumour stage (Table 5). Intriguingly, there was a significant preponderance of medium- and high-expressing tumour samples in men compared with women (*P*=0.03). In an univariate analysis, intensity of aberrant expression of ADAM17 was not significantly correlated to overall survival (high: median 10.9 months, medium: median 22.8 months and low: median 16.3 months, *P*=0.09, Figure 3C, Table 3) or progression-free survival (high: median 9 months, medium: median 17 months and low: median 14 months, *P*=0.43).

## Discussion

Our study for the first time shows that the increased cytoplasmic expression of ALCAM is an independent prognostic marker for early tumour relapse and poor OS in pancreatic cancer. From a functional point of view, this correlation could be explained by the pro-tumourigenic capacities of ALCAM. Jezierska *et al* (2006) discovered that ALCAM protected breast cancer cells against apoptosis and autophagy. Choi *et al* (2000) showed that in fibrosarcoma cell lines ALCAM augmented chemoresistance and enhanced the metastatic potential. Lunter *et al* (2005) showed that ALCAM regulated matrix metalloproteinase-2 in melanoma cell lines, hence contributing to invasive tumour growth. In analogy, one can speculate that the accumulation of ALCAM in pancreatic tumour cells leads to similar tumour-promoting effects, therefore having a negative prognostic impact.

However, our results as well as other studies are conflicting if taking into account that reduced expression of ALCAM has also been related to poor survival in some types of cancer (King *et al*, 2004; Mezzanzanica *et al*, 2008). Mezzanzanica *et al* (2008) postulated that decreased expression of ALCAM in ovarian cancer results from ALCAM delocalisation from the cell membrane to cytoplasm, thus increasing the migratory properties of malignant cells. In our study, we could show that in normal pancreatic tissue, ALCAM is predominantly expressed in the cellular membrane, whereas in pancreatic tumour cells, it is mainly localised in the cytoplasm. Intriguingly, however, in the small subset of our clinical specimen, exhibiting partly membranous expression in addition to cytoplasmic expression, progression-free survival and OS was significantly reduced in univariate analysis compared with patient samples without any membranous expression. These results are in accordance with previous findings in colorectal cancer (Weichert *et al*, 2004), but contradict the assumption that the translocation of ALCAM from the cellular membrane into the cytoplasm is its only pivotal mechanism contributing to tumour promotion, and therefore is associated with an adverse prognosis.

All in all, it still remains unanswered and a challenge to further investigate (i) why in the majority of cancer entities examined so far, overexpression of ALCAM is a negative prognostic marker, whereas in some other tumour types its downregulation is associated with an unfavourable prognosis and (ii) which role plays the differential cellular localisation of ALCAM in tumour progression and metastasation.

To obtain some deeper insight in the last mentioned question, we examined a potential co-expression of ADAM17 and ALCAM in pancreatic cancer. ADAM17 has been discovered to be an important proteolytic sheddase of ALCAM (Bech-Serra *et al*, 2006). This mechanism can enhance tumour cell motility and migratory capacity, hence increasing the invasive and metastatic potential (Rosso *et al*, 2007). We could show that ADAM17 is significantly overexpressed in pancreatic cancer compared with normal pancreatic tissue. These findings in a large collective are consistent with previous results including a smaller size of samples (Ringel *et al*, 2006). But contrary to Ringel *et al*, we observed ADAM17 mainly occurring in the cell cytoplasm and not in the cellular membrane. These different observations are difficult to explain, as we and Ringel *et al* employed similar methods of immunohistochemistry and used the same type of ADAM17-antibody. Yet, as our data are obtained from a much larger collective of patient samples, they seem to be more valid by statistical considerations. This study is the first to evaluate ADAM17 as potential prognostic marker in a large number of clinical specimens in pancreatic cancer. However, despite the undoubtfully important role of ADAM17 in tumour disease (Arribas *et al*, 2006; Ringel *et al*, 2006), our results cannot prove ADAM17 to be a prognostic marker in pancreatic cancer. Nor, we could observe a relevant co-expression between ALCAM and ADAM17. Thus, to answer to what extent the proteolytic sheddase of ALCAM by ADAM17 is a relevant step for tumour progression in pancreatic cancer, further experimental studies are required.

Taken together, our data confirm the relevance of ALCAM as an important predictor for clinical outcome in cancer. Learning more about its biological functions in pancreatic cancer will help to gain new insights into the pathogenesis of pancreatic cancer.

## Figures and Tables

**Table 1:** ALCAM displays a differential cellular expression pattern in pancreatic cancer and normal peritumoral pancreatic tissue (*χ*^2^-test)

	**Cellular localisation**			
	**Negative staining**	**Cytoplasmic staining**	**Membranous staining**	***P*-value**
Pancreatic cancer (*n*=97)	41	50	6	
Normal pancreatic tissue (*n*=60)	12	0	48	0.0001

ALCAM=activated leucocyte cell adhesion molecule.

**Table 2:** Correlation between CD166 expression and clinical and pathological parameters (*χ*^2^-test)

**Characteristics**	**Number of cases**	**CD166 low**	**CD166 medium**	**CD166 high**	***P*-value**
Total	97	67	23	7	
					
*Age*					
<Mean	46	33	9	4	0.61
⩾Mean	51	34	14	3	
					
*Gender*					
Female	44	33	10	1	0.20
Male	53	34	13	6	
					
*Lymph node status*					
N0	18	12	4	2	0.78
N1	79	55	19	5	
					
*Grading*					
1	4	4	0	0	0.34
2	57	42	12	3	
3	36	21	11	4	
					
*AJCC tumour stage*					
2	18	12	4	2	0.59
3	74	50	19	5	
4	5	5	0	0	
					
*R-status*					
	88	59	22	7	0.38
	9	8	1	0	
					
*Survival status after 36 months* a					
Deceased	64	40	18	6	**0.03**
Alive	27	24	3	0	

AJCC=American Joint Committee on Cancer.

a Only patients with complete follow-up included. Significantly difference values are indicated in bold.

**Table 3:** Univariate analysis (log-rank test) of prognostic parameters in pancreatic cancer for median progression-free (PFS) and median overall survival (OAS)

	**PFS** a			**OAS** b		
**Characteristics**	**Number of cases**	**Time (months)**	***P*-value**	**Number of cases**	**Time (months)**	***P*-value**
*Adjuvant chemotherapy* c						
Not received	14	7	0.6	19	4.5	**0.0001**
Received	65	15		69	22.8	
						
*Lymph node status*						
N0	16	24	0.34	17	32.2	**0.02**
N1	63	12		75	16.3	
						
*Grading*						
1	4	36	0.13	4	37.3	0.33
2	48	15		53	18.5	
3	27	11		35	15.5	
						
*AJCC tumour stage*						
2	16	24	0.3	17	32.2	**0.01**
3	58	12		70	17.7	
4	5	6		5	10.9	
						
*Resection status*						
R0	72	24	0.77	83	18.7	0.95
R1	7	15		9	13.6	
						
*CD166*						
Low	57	17	**0.008**	65	22.8	**0.0006**
Medium	19	8		21	14.8	
High	3	7		6	5.6	
Membranous	6	6	**0.02**	6	10.9	**0.03**
Non-membranous	73	15		86	18.7	
						
*ADAM17*						
Low	34	14	0.43	40	16.3	0.09
Medium	34	17		40	22.8	
High	11	9		12	10.9	

AJCC=American Joint Committee on Cancer

a Total number of cases only 79, in 12 cases exact time of tumour recurrence could not be specified

b New: total number of cases only 92, five cases lost to follow-up),

c Type of adjuvant treatment in four cases unknown. Significantly difference values are indicated in bold.

**Table 4:** Multivariate analysis (Cox proportional hazards regression model, non-categorial) of prognostic parameters for overall survival in pancreatic cancer

**Characteristics**	***β***	**Standard-error**	**Wald**	**d.f.**	**Relative risk**	**95% CI of relative risk**	***P*-value**
Adjuvant chemotherapy *vs* no adjuvant chemotherapy	−1.889	0.295	41.127	1	0.151	0.085–0.269	**0.0001**
CD 166 expression low *vs* medium/high	1.053	0.270	15.166	1	2.866	1.687–4.869	**0.0001**
Tumour stage AJCC II *vs* III/IV	0.999	0.367	7.396	1	2.716	1.322–5.581	**0.007**

AJCC=American Joint Committee on Cancer; d.f.=degree of freedom. Significantly difference values are indicated in bold.

**Table 5:** Correlation between ADAM17 expression and clinical and pathological parameters (*χ*^2^-test)

**Characteristics**		**ADAM17 low**	**ADAM17 medium**	**ADAM17 high**	***P*-value**
Total	97	43	40	14	
					
*Age*					
<Mean	46	19	18	9	0.39
⩾Mean	51	24	22	9	
					
*Gender*					
Female	44	26	13	5	**0.03**
Male	53	17	27	9	
					
*Lymph node status*					
N0	18	6	9	3	0.58
N1	79	37	31	11	
					
*Grading*					
1	4	2	2	0	0.66
2	57	24	26	7	
3	36	17	12	7	
					
*AJCC tumour stage*					
2	18	6	9	2	0.39
3	74	35	30	9	
4	5	2	1	2	
					
*R-status*					
	88	38	36	14	0.42
	9	5	4	0	
					
*Survival status after 36 months* a					
Deceased	64	29	25	10	0.49
Alive	27	11	14	2	

AJCC=American Joint Committee on Cancer

a Only patients with complete follow-up included. Significantly difference values are indicated in bold.
