# Supplementary material for: Increased expression of ALCAM/CD166 in pancreatic cancer is an independent prognostic marker for poor survival and early tumour relapse
Source: Br J Cancer. 2009 Jul 14;101(3):457–64. doi: 10.1038/sj.bjc.6605136 (PMC2720248; doi:10.1038/sj.bjc.6605136)
Supplement: Supplementary Figures Legends [file 6605136x18.doc]

Supplementary Figure 1 c: ALCAM expression in pancreatic cancer. Squarish cutout displays Figure 1 c in low magnification.

Supplementary Figure 2 b: ADAM17 expression in pancreatic cancer. Squarish cutout displays Figure 2 b in low magnification.

Supplementary Figure 2 c: ADAM17 expression in pancreatic cancer. Squarish cutout displays Figure 2 c in low magnification.

Supplementary Figure 2 d: ADAM17 expression in pancreatic cancer. Squarish cutout displays Figure 2 d in low magnification.

Supplementary Figure 4 a – f: Comparative immunohistochemical analysis of primary rabbit polyclonal anti-ALCAM antibody (a, c, d) and primary mouse monoclonal anti-ALCAM antibody in pancreatic cancer (b, d, f).

Supplementary Figure 4 g: ALCAM expression in pancreatic cancer (polyclonal anti-ALCAM antibody (Abnova, Taiwan)).

Supplementary Figure 5 a – f: Comparative immunohistochemical analysis of primary rabbit polyclonal anti-ADAM17 antibody (a, c, d) and primary mouse monoclonal anti-ADAM17 antibody in pancreatic cancer (b, d, f).
